# Enhanced secondary analysis of survival data: reconstructing the data from published Kaplan-Meier survival curves

**DOI:** 10.1186/1471-2288-12-9

**Published:** 2012-02-01

**Authors:** Patricia Guyot, AE Ades, Mario JNM Ouwens, Nicky J Welton

**Affiliations:** 1 School of Social and Community Medicine, University of Bristol, Canynge Hall, 39 Whatley Road, Bristol BS8 2PS UK; 2 Mapi Consultancy, De Molen 84, 3995 AX Houten, the Netherlands

**Keywords:** Survival analysis, Individual Patient Data, Kaplan-Meier, algorithm, life-table, Cost-Effectiveness Analysis, Health Technology Assessment

## Abstract

**Background:**

The results of Randomized Controlled Trials (RCTs) on time-to-event outcomes that are usually reported are median time to events and Cox Hazard Ratio. These do not constitute the sufficient statistics required for meta-analysis or cost-effectiveness analysis, and their use in secondary analyses requires strong assumptions that may not have been adequately tested. In order to enhance the quality of secondary data analyses, we propose a method which derives from the published Kaplan Meier survival curves a close approximation to the original individual patient time-to-event data from which they were generated.

**Methods:**

We develop an algorithm that maps from digitised curves back to KM data by finding numerical solutions to the inverted KM equations, using where available information on number of events and numbers at risk. The reproducibility and accuracy of survival probabilities, median survival times and hazard ratios based on reconstructed KM data was assessed by comparing published statistics (survival probabilities, medians and hazard ratios) with statistics based on repeated reconstructions by multiple observers.

**Results:**

The validation exercise established there was no material systematic error and that there was a high degree of reproducibility for all statistics. Accuracy was excellent for survival probabilities and medians, for hazard ratios reasonable accuracy can only be obtained if at least numbers at risk or total number of events are reported.

**Conclusion:**

The algorithm is a reliable tool for meta-analysis and cost-effectiveness analyses of RCTs reporting time-to-event data. It is recommended that all RCTs should report information on numbers at risk and total number of events alongside KM curves.

## Background

Normal practice in the reporting of results from RCTs is to publish the sufficient statistics for each arm: means and standard deviations for continuous outcomes, numerators and denominators for binary outcomes. CONSORT guidelines recommend that for each primary and secondary outcome "study results should be reported as a summary of the outcome in each group, together with the contrast between the groups, known as the effect size" [1]. The publication of sufficient statistics facilitates the inclusion of the trial in subsequent meta-analysis or economic assessments. However, reporting results of trials with survival time outcomes almost never follows these principles [2]. Due to censoring, time-to-event outcomes are not amenable to standard statistical procedures used for analysis of continuous outcomes: the average survival time is a biased estimate of expected survival in the presence of censored observations. Consort guidelines recommend instead, that for survival time, the measure of effect could be the hazard ratio or difference in median survival time. The Cochrane handbook also advises that the effect measure for time-to-event outcomes should be expressed as a hazard ratio. This severely limits the way in which RCTs with survival time data can be included in any kind of secondary data analyses, whether as part of a cost-effectiveness analyses (CEA) or in an analysis of treatment efficacy.

In the case of CEA, what is required is an estimate of the difference between arms in expected survival: this cannot be reconstructed from the reported, or pooled, hazard ratio or medians without further assumptions. This demands that the survival curves are extrapolated for each treatment with a lifetime horizon, which is clearly impossible based solely on a hazard ratio or a median estimate.

For the evidence syntheses, i.e. meta-analyses or network meta-analyses, pooling the treatment effect over several trials must either use estimates of median survival, which has been shown to be unsatisfactory [3], or fall back on estimates of the hazard ratio. This is also limiting and unsatisfactory, as it requires proportional hazards, an assumption that is seldom checked and sometimes implausible on inspection. These issues are examined further in the discussion.

As a response to the poor, limited, and inconsistent reporting of results from survival data, several authors have attempted to extract data from the published Kaplan-Meier (KM) curves in order to carry out meta-analysis [4-11]. However, in this earlier work the survival probabilities were extracted from the graphs or the text at a relatively small number of follow-up times in order to approximate aggregate or life-table data, and they did not use all of the information reported to help identify the censoring pattern.

In this paper, we develop an algorithm that attempts to reproduce the sufficient statistics in detail, by reconstructing the Kaplan-Meier data on which the survival curves are based. The KM curves are in effect pictorial representations of these data. We use digital software to read in the coordinates of the KM curves from the published graph and we use the information on numbers at risk, often published at four or five time points under the x-axis of the KM graph, and total number of events, where available, to reconstruct the Kaplan-Meier data for each arm. A key feature of our approach is the use of iterative numerical methods to solve the inverted KM equations, which is necessary to obtain consistent results and make the best use of the information available.

The paper is organised as follows. In the illustration section, we apply the algorithm to one published Kaplan-Meier curve. In the reliability and accuracy section, we compare the summary measures reported in the original publications with those obtained by the analysis of the reconstructed Kaplan-Meier data on six pairs of KM curves reconstructed by three observers each on two occasions. We conclude with a discussion of the potential of this technique in meta-analysis and health technology assessment based on cost-effectiveness analysis. In the method section, we briefly describe the Kaplan-Meier estimation method. We then describe the inputs required for the algorithm, before presenting the algorithm itself. The general principle of the algorithm is developed as well as solutions to some particular pitfalls that may be encountered in practical applications. The R-code for the algorithm is provided in the Additional file 1.

## Results

### Illustration

**Figure 1:**
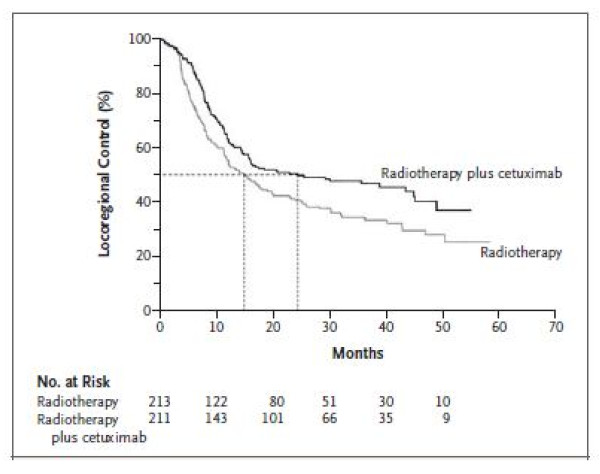
**Example of published Kaplan-Meier curves **[13].

The illustrative example uses KM curves, reproduced in Figure 1, on locoregional control events in head and neck cancer [12]. The numbers at risk were available every 10 months, from zero to fifty months. There was no total number of events reported in the publication.

The tables 1 and 2 illustrate the input data needed to run the algorithm for this figure. Table 1 shows the extracted co-ordinates for the arm 'radiotherapy' between 0 and 10 months using the DigitizeIt software. Table 2 presents the format required for the numbers at risk provided in the publication. The first column is the interval, the second column shows the time, the third column shows the row of the extracted co-ordinates that the time corresponds to, the fourth column is the upper row of the extracted co-ordinates for which the time is less than the following time at which we have a number at risk and the last column is the number at risk. Time 0 always corresponds to the 1^st ^row. In this example, row 30 corresponds to the largest time extracted from the graph-reading software that is less than 10 months. Time 10 corresponds to row 31 (i.e. the 31^st ^"click" from the graph-reading software). And so on.

**Table 1:** Example of x-axis (time) and y-axis (Locoregional control) co-ordinates extracted with DigitizeIt (corresponding to Figure 1, radiotherapy arm, between 0 and 10 months)

Extracted co-ordinate, k	Time in months, Tk	Locoregional control, Sk
1	0	1
2	0.18	0.994
3	0.42	0.989
4	0.91	0.979
5	1.39	0.974
6	1.88	0.969
7	2.6	0.964
8	2.85	0.959
9	3.33	0.933
10	3.34	0.923
11	3.58	0.901
12	3.83	0.865
13	4.07	0.85
14	4.56	0.828
15	4.8	0.817
16	5.29	0.777
17	5.54	0.767
18	5.78	0.759
19	6.02	0.749
20	6.51	0.716
21	6.75	0.711
22	7.73	0.671
23	7.97	0.661
24	8.21	0.651
25	8.46	0.638
26	8.7	0.628
27	8.7	0.626
28	8.95	0.621
29	9.43	0.616
30	9.92	0.61
31	10	0.608

**Table 2:** Example of number at risk file (corresponding to Figure 1, radiotherapy arm)

Interval, i	**Time in months, trisk**_**i**_	**Lower, lower**_**i**_	**Upper, upper**_**i**_	**Number at risk, nrisk**_**i**_
1	0	1	30	213
2	10	31	58	122
3	20	59	83	80
4	30	84	102	51
5	40	103	120	30
6	50	121	128	10

**Figure 2:**
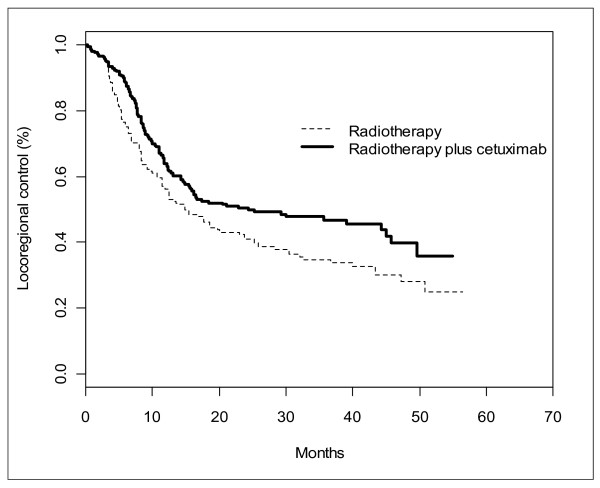
**Example of reconstructed Kaplan-Meier curves**.

Using the algorithm, we obtain reconstructed IPD and it allows us to reconstruct the KM curves as shown in Figure 2. The original publication reported, in addition to the KM curves, the following summary results: the survival rates at one, two and three years, the median duration and the hazard ratio with its uncertainty. These published summary results and their corresponding reconstructed summary results are presented in Table 3.

**Table 3:** Example of summary measures collected from the original publication example [7] and their corresponding estimates obtained from the reconstructed IPD

	Original publication	Reconstructed IPD
**Radiotherapy arm**		

survival rate (1 year)	55	56.1 (49.6; 63.3)

survival rate (2 years)	41	41.1 (34.7; 48.6)

survival rate (3 years)	34	34.7 (28.4; 42.5)

median duration	14.9	14.9 (11.9; 23.0)

**Radiotherapy plus cetuximab arm**		

survival rate (1 year)	63	64.0 (57.8; 70.9)

survival rate (2 years)	50	50.4 (43.9; 57.8)

survival rate (3 years)	47	46.7 (40.1; 54.4)

median duration	24.4	24.3 (15.7; 45.7)

**Hazard ratio with 95%CI**		

	0.68 (0.52; 0.89)	0.73 (0.57; 0.94)

### Reproducibility and accuracy of the algorithm

The validation results are summarised in Table 4.

**Table 4:** Reproducibility and accuracy results for the survival probabilities, the medians, the HRs and their uncertainties; ME: mean error; MAE: mean absolute error; *σ*_*r *_: standard deviation due to reproducibility; *σ*_*e *_: standard deviation due to exemplar

Survival probabilities				
	ME (95%CI)	MAE (95% CI)	***σ***_***r ***_**(95% CI)**	***σ***_***e ***_**(95% CI)**
All information	-0.103% (-0.260; 0.055)	0.272% (0.021; 1.544)	0.270% (0.234; 0.577)	0.226% (0.138; 0.410)

No numbers at risk	-0.051% (-0.186; 0.083)	0.279% (0.019; 1.321)	0.294% (0.253; 0.411)	0.205% (0.113; 0.383)

No total events	0.079% ( -0.111; 0.269)	0.358% (0.035; 2.504)	0.316% (0.283; 0.600)	0.396% (0.293; 0.579)

Neither	0.101% (-0.069;0.270)	0.328% (0.031; 2.233)	0.289% (0.259; 0.547)	0.373% (0.277; 0.547)

**Medians**				
	ME (95%CI)	MAE (95% CI)	***σ***_*r *_(95% CI)	***σ***_*e *_(95% CI)

All information	0.011 (0.004; 0.018)	0.011 (0.001; 0.036)	0.006 (0.004; 0.012)	0.005 (0.001; 0.037)

No numbers at risk	0.010 (0.005; 0.014)	0.010 (0.001; 0.027)	0.006 (0.005; 0.021)	0.002 (0.000; 0.021)

No total events	0.005 (-0.001; 0.012)	0.010 (0.001; 0.045)	0.011 (0.009; 0.015)	0.008 (0.003; 0.019)

Neither	0.004 (-0.001; 0.010)	0.011 (0.001; 0.045)	0.015 (0.012; 0.021)	0.005 (0.000; 0.016)

**Hazard ratios**				
	ME (95%CI)	MAE (95% CI)	***σ***_*r *_(95% CI)	***σ***_*e *_(95% CI)

All information	0.008 (-0.015; 0.030)	0.017 (0.002; 0.122)	0.021 (0.017; 0.041)	0.021 (0.009; 0.085)

No numbers at risk	0.007 (-0.036; 0.049)	0.036 (0.003; 0.242)	0.037 (0.028; 0.058)	0.041 (0.019; 0.164)

No total events	0.021 (-0.004; 0.045)	0.028 (0.002; 0.167)	0.018 (0.015; 0.029)	0.029 (0.017; 0.074)

Neither	0.037 (-0.190; 0.264)	0.198 (0.021; 1.556)	0.016 (0.013; 0.028)	0.284 (0.177; 0.699)

**Standard errors of the log hazard ratios**				
	ME (95%CI)	MAE (95% CI)	***σ***_*r *_(95% CI)	***σ***_*e *_(95% CI)

All information	0.002 (-0.035; 0.040)	0.021 (0.002; 0.149)	0.024 (0.017; 0.114)	0.023 (0.005; 0.764)

No numbers at risk	-0.010 (-0.034; 0.014)	0.016 (0.001; 0.095)	0.016 (0.012; 0.060)	0.016 (0.002; 0.526)

No total events	-0.022 (-0.057; 0.014)	0.033 (0.003; 0.204)	0.018 (0.014; 0.055)	0.035 (0.019; 0.133)

Neither	-0.143 (-0.262; -0.023)	0.143 (0.010; 0.736)	0.039 (0.031; 0.100)	0.121 (0.067; 0.452)

Note that the study 1 did not report the total number of events.

#### Survival probabilities

Using full information, we found a mean error of -0.103% (95%CI:-0.260; 0.055). This means that if the original survival probability estimate was 50%, we would expect survival probability based on reconstructed data to be 49.897% (95% CI: 49.740: 50.055). There is therefore no significant systematic error. The mean absolute error (MAE) is 0.272%. Thus, if the original estimated survival was 50% we would expect any estimate to be 0.272% on either side (i.e. 49.728 or 50.272), with a 95% CI going up to 1.544%. Thus 97.5% of the time the error would be less than 1.544%. The reproducibility standard deviation was 0.270% (95% CI: 0.234, 0.577). One way to consider this is that about 68% of observations will be within 0.270% of their mean value, either way. The variation due to exemplar differences is of the same order.

As the level of information available is decreased by successively removing data on numbers at risk and number of events, the ME and the reproducibility standard deviation remain unaltered. There is, however, a slight fall in accuracy as assessed by MAE and exemplar variance.

#### Medians

The ME on the log scale was 0.011 (95%CI: 0.004; 0.018). By taking the exponentials of these values, we obtain that the mean error is on average a factor of exp (0.011), or 1.1% (95%CI: 0.4%; 1.8%). The mean error is statistically different from zero, but still extremely small. For example, if the median survival in the original data was reported as 2 years, the expected median in the reconstructed data would be 2.022 years (95%: 2.008, 2.036). The MAE error is of the same order. Reproducibility variation is also exceptionally low at 0.006 on the log scale, corresponding to a 0.6% geometric standard deviation. Thus, we would expect that 68% of the observations are within 0.6% (95% CI: 0.4%; 1.2%) either side of the original median. Similar results are seen at all the levels of information.

#### Hazard ratios

With full information we obtained a ME on the log scale of 0.008 (95%CI:-0.015; 0.030). By taking the exponentials again, we can infer that if the original HR is 1.5, or 0.667 for its inverse, then we would expect to obtain a reconstructed HR of 1.512, or 0.661 for its inverse. The confidence intervals for the ME span zero, indicating no statistically significant systematic error. Looking at the MAE of 0.017 (95%CI: 0.002; 1.222), we can infer that if the original HR was 1.5, or 0.667 for its inverse, we would expect the reconstructed HR would be within a factor or exp (0.017) = 1.017 either side of the original values, i.e. 1.475 or 1.525, or 0.656 or 0.678 for its inverse. Based on the upper confidence limit we would expect 97.5 of reconstructed HRs to be within a factor of exp (0.122) = 1.13 either side of the original values: for an original value of 1.5, or 0.667 for its inverse, this means that 97.5% of reconstructed values will be between 1.33 and 1.69, or between 0.59 and 0.75 for its inverse. With full information the reproducibility is good: 68% of values are expected to be within exp (0.021) = 1.02 of their mean value, or 1.04 if we take the upper confidence limit. The variation due to choice of exemplars is of similar magnitude to the MAE.

In contrast to survival probabilities and medians, both the MAE and the exemplar variance deteriorate markedly as less and less information is provided. If neither numbers at risk nor number of events is available, then for an original HR of 1.5, or 0.667 for its inverse, we would expect the reconstructed HR to be within a factor of exp (0.198) = 1.2 either side of the original value, i.e. 1.23 or 1.83, or 0.55 or 0.81 for its inverse. The upper confidence limit would allow as much as a factor of exp (1.556) = 4.7 on either side of the original, in other words reconstructed HRs as low as 0.32 or as high as 7.11 for an original HR of 1.5, or as low as 0.14 or as high as 3.16 for its inverse.

#### Standard errors of the log hazard ratios

The ME was not significantly different from zero when using full information. The ME on the log scale was estimated to be 0.002 (95%CI: -0.035; 0.040). In the 'neither' case, the ME became significantly negative, meaning that the uncertainty in the reconstructed HRs was underestimated. This is due to the assumption of no censoring which was made in this case.

## Discussion

For CEA, an estimate of expected (mean) survival time is needed, therefore reported or pooled HRs are clearly insufficient. A reliable analysis can only be conducted if access to IPD for each source of efficacy evidence is available. This allows investigation of a range of parametric models and selection of an appropriate model for the underlying distribution and for the treatment effect.

Unfortunately, IPD is only rarely available, particularly if the secondary analysis is to be a meta-analysis of several trials or where several different treatments have been examined. If a meta-analysis of time-to-event-outcomes is being considered for use in a CEA, this should be an IPD meta-analysis.

There is a strong case to be made that the same statistical model should be used for both efficacy and CEA analyses [13]. But even if the secondary analysis is focussed purely on efficacy, it is noteworthy that IPD meta-analysis of time-to-event outcomes has been described by many authors as the gold standard [5,6,11,14]. One reason for this is that it allows a more informative analysis of time-dependent data [15]. First, aggregated results from time-to-event trials may be reported as medians or as HRs, but it is hard to combine trials reporting medians with trials reporting HRs without making distributional assumptions. Secondly, if HRs are reported one is obliged to accept the proportional hazards assumption, even though this is seldom checked [13] in the primary analysis. Third, although it is often believed that PH provides an approximate "average" HR in cases where PH does not hold, such estimates are clearly vulnerable to bias. If, for example, the HR is diminishing over time, the procedure will over-estimate the HR whenever the trials differ in follow-up time. Finally, even in the best possible case for meta-analysis based on HRs, where every study reports the HR *and *every study tests the null hypothesis of PH and fails to reject it, it should be remembered that trials are not powered to detect departure from PH [16], and a superior test of PH can always be achieved by an IPD meta-analysis of the entire set of trials.

The algorithm suggested here allows investigators to re-create KM data, allowing them to explore the proportional hazards assumption, and freeing them to fit a richer class of survival distributions to the reconstructed data. Access to the IPD, or to reconstructed data, allows a huge liberalization in modelling survival. Multiple parameter distributions can be implemented [17-19], or flexible spline approaches [20]. Methods to adjust for "cross-over" of treatment can also be applied [21]. In the context of synthesis, treatment effects can be put on shape and scale parameters [8] or fractional polynomials can be used [9].

The validation exercise established that reproducibility and accuracy of reconstructed statistics was excellent, especially for median survival and probability of survival. In addition these reconstructed statistics were relatively insensitive to deterioration in the level of information used. Reproducibility and accuracy of reconstructed Hazard Ratios was less good but certainly adequate with complete information, but became increasingly inaccurate with less information, and frankly unusable when neither numbers at risk nor number of events were available.

The reason why the HR is generally reconstructed with less accuracy and more vulnerable to the level of information is because it is in effect a weighted average of ratios along the entire risk period, while survival probabilities and medians are simple point estimates. The reconstruction algorithm must make assumptions about the degree of censoring within each segment and these assumptions must affect the relative weighting of different portions of the curve. As the level of information is reduced, the assumptions become increasingly unrealistic. As it has been noted previously, though in a slightly different context [22], we might anticipate that reconstructed HRs will be less accurate in cases where the data departs more from proportional hazards. It should be emphasised that our purpose in reconstructing the KM data is not to obtain an estimate of the HR, when it is not reported, but instead to allow investigators access to a good approximation of the Kaplan-Meier statistics.

Previous work using published KM curves in secondary analysis have approached the issue in several different ways [4-11]. In most cases, it is clear that the information has been extracted from the curves [6-11], but only a few authors [8-10] report that they carry out the data extraction using digitizing software. Dear [4] extracts the survival probabilities and their variance and estimates their covariance using normal approximations under the assumption of no censoring. Arends [5] adopts a similar strategy but uses a complementary log log link to model the probabilities. Earle [10] provides a comparison of several methods [4,23-25] by extracting the survival probabilities and estimating the number of patients at risk and number of events in successive time intervals by using the actuarial equations, using information on censoring if reported and ignoring censoring otherwise. Williamson [11] uses the survival probabilities and the numbers at risk provided below the curves to estimate the number of events on the intervals defined by the numbers at risk provided, using the actuarial method. Parmar's method [7], frequently cited and used in the meta-analysis literature, uses information on the minimum and maximum of follow-up to inform the censoring pattern, in addition to the extracted survival probabilities, to estimate numbers of events and numbers at risk in successive time intervals, and then produce estimates of hazard ratios in cases where these are not available in the published papers. Fiocco [6] assumes a Poisson distribution and uses log-linear modelling based on estimated data using the same approach as Parmar. Finally, Ouwens [8] and Jansen [9] digitise the KM graphs at particular time points and use the number at risk at the beginning of the interval, if this is reported, or conservatively assume no censoring if it is not. The life-table data is then reconstructed as a series of conditionally independent binomial distributions.

The primary objective of this previous work was neither the reconstruction of Kaplan Meier data nor the reconstruction of life-table data, but was a necessary step that had to be taken in order for the authors to illustrate methods for combining survival data from several studies. All these previous attempts reconstructed the data in the form of life-table data at a limited number of time points, whereas we have tried to reconstruct the original KM intervals. Published survival curves are almost always based on the KM method justifying our approach of using inverted KM equations instead of life-table equations and solving at the same time the problem of pre-specification of intervals. For the 'all information' and 'no total events' cases, the censoring pattern varied by numbers at risk published intervals as in Williamson [11]. For the 'no number at risk' case, the censoring pattern is assumed constant over the interval and for the 'neither' case, no censoring is assumed. Except for the 'neither' case where there is no information on number at risk or total number of events, we believe that our method represents an improvement over the other approaches described above. This is because we used an iterative numerical approach to solve the inverted KM equations, not described elsewhere, which is required to ensure consistency between the successive estimates of numbers at risk and reported values, and/or estimate of total number of events and reported value. Without such a procedure, it is not possible to make the change in survival probabilities over an interval and number of events and censorings within the interval consistent with the number at risk at the start of the next interval. We therefore believe that the methods proposed here are likely to be the most accurate of the methods proposed so far, when at least numbers at risk or total events are reported.

Very few authors [7,10] reported any assessment of their reconstruction of data. Earle [10] evaluated the reproducibility of using digitized software to extract the data by reporting an intraclass correlation coefficient. Parmar [7] calculated the ME on 48 studies between reconstructed HR using published survival curves and reconstructed HR using more direct estimates from either the Cox model or the logrank test results. He found no systematic bias for the HR and a slight systematic underestimation for their variance. We have provided a more complete evaluation of our method by reporting not only good reproducibility and lack of systematic error, but also information on MAE which tells us about the accuracy of the reconstruction that can be expected in a given instance.

Limitations of the new method should be mentioned. To the extent that published KM curves tend to pool data over different covariates that might affect survival, the method is still not quite the same as having true IPD. The inability to derive separate KM curves for different subgroups or to model the joint effects of covariates and treatment can impact on the estimated treatment effect due to aggregation bias [26]. Even if the arms are well balanced on a covariate, and even if the covariate is not an effect-modifier, aggregation over the covariate will tend to bias the treatment effect towards the null, and the extent of the bias increases with the strength of the covariate effect. However, this is an issue for all meta-analysis where a covariate adjustment could not been performed.

A second limitation is that the reliability of the reconstructed data depends on two related elements: the quality of the initial input and the level of information provided by the publication. The figure extracted from the .pdf should not be of low quality (for instance, blurry figure and/or poor numerical axis scale); otherwise the user may struggle to extract accurate data via the digitising software. Furthermore, the extracted data needed to run the algorithm should be consistent and sufficient. We encourage users to undertake the initial digitization and pre-processing with scrupulous care. In our experience, although little training is required, it takes about half an hour to obtain the initial input for one curve. If incorrect data are entered this may trap the algorithm. The algorithm has been developed to be used for the large amount of RCTs reporting KM curves. However, as we have seen in the results section, if the total number of events and the numbers at risk other than at time zero are not provided, the algorithm may produce poor results. We recommend that all the information available is used, and wherever possible the reconstructed KM data should be compared with all results (survival probabilities, medians, hazard ratio) reported in the original publication. We recommend that all RCTs with time-to-event outcomes publish KM curves together with information on numbers at risk and total number of events.

## Conclusion

The objective of this article was to present a reliable algorithm permitting the reconstruction of data based on KM curves reported in the literature. The algorithm is implemented with the help of a few basic equations. The complete program is available in the appendix in the R syntax. This method has the potential to transform secondary analysis of survival data, whether for CEA or efficacy analysis.

## Methods

### The Kaplan-Meier (KM) estimation method

The Kaplan-Meier (KM) method is used to estimate the probability of experiencing the event until time *t, S*^*KM *^(*t*), from individual patient data obtained from an RCT that is subject to right-censoring (where some patients are lost to follow-up or are event-free at the end of the study period). The method works by summarising the IPD in the form of a series of *r *time intervals [0, *t*_1_), [*t*_1_, *t*_2_),..., [*t*_*r*_, ∞). These intervals are designed to be such that at least one event occurs at the start of each interval. For each time interval *m *= 1, 2,.., *r*, the Kaplan-Meier data consist of the number of events that occur at the start of the interval, *d*_*m*_, the number of individuals censored on the interval, *c*_*m*_, and the number of patients still at risk just before the start of the interval, *n*_*m*_, so that

(1) $$n_{m+1}=n_{m}-d_{m}-c_{m}.$$

The Kaplan-Meier data then provide the sufficient statistics required to form the Kaplan-Meier estimate of the survival function [17]*S*^*KM *^(*t*_*m*_) at event time *t*_*m*_:

(2) $$\begin{array}{c} S^{KM}\left(t_{m}\right)=\overset{m}{\underset{j=1}{\prod }}\frac{n_{j}-d_{j}}{n_{j}}=S^{KM}\left(t_{m-1}\right)*\frac{n_{m}-d_{m}}{n_{m}}\\ m=1,2,\ldots ,r \end{array}$$

### The Kaplan-Meier data reconstruction algorithm

#### Data inputs required

The first input data file required for the algorithm contains the extracted x-axis coordinates, *T*_*k*_, and y-axis coordinates, *S*_*k*_, for *k *= 1,..., *N *points on the KM curve. Several software packages exist to do this, and we found that the software DigitizeIt (http://www.digitizeit.de/) performed well. The KM curves, extracted from a .pdf article, are read into the software, the axes are defined, and then the analyst uses mouse-clicks to select points to read off from the curve. The resulting *T*_*k *_and *S*_*k *_coordinates are then exported into a text file. This preliminary work needs to be performed carefully. The data should be sufficient: every step seen in the figures should have been captured during the data extraction. The location and the number of clicks are therefore important. The data should also be consistent: the probability of experiencing the event decreases with time, and it should be verified that this is always the case for the data points extracted. Anomalies may occur due to the publication quality of the curve, and human error in controlling the clicks. Any anomalies should be corrected before running the algorithm below. The times, at which the numbers at risk are reported in the publication, must be included in these initial data. As a convention, the first data point is *T*_1 _= 0 and probability of experiencing the event to time 0 is therefore *S*_1 _= 1. Each KM curve is extracted separately.

The second input data file required for the algorithm contains information on the reported numbers at risk. The curve is split into *i = 1,.., nint *intervals, for each we have the reported number at risk at the start of that interval, *nrisk*_*i*_, the time at which the number at risk is provided, *trisk*_*i*_, the first row number of the extracted co-ordinates for that time interval *lower*_*i*_, and the last row number of the extracted co-ordinates for that time interval *upper*_*i*_*. nrisk*_*i *_and *trisk*_*i *_come from the original publication, while *lower*_*i *_and *upper*_*i *_come from the number of clicks done on each interval, in order to create the first input data file. For each *i, lower*_*i *_is equal to *k *when *T*_*k *_= *trisk*_*i *_and *upper*_*i *_is equal to *k *when *T*_*k*+1 _= *trisk*_*i*+1_.

The final input data required is the total number of events, *totevents*.

We begin by describing the algorithm for the case where the number at risk is reported at the start of the study and at least one other time-point and when the total number of events is reported ('*all information' *case). We then show how the algorithm can be adapted when the number at risk is only reported at the beginning of the study (*'no numbers at risk' *case), when the total number of events is not reported (*'no total events' *case), and when neither of these are reported (*'neither' *case).

#### The algorithm for the 'all information' case

The number of censored individuals is not available from the reported data. We therefore use the reported numbers at risk, *nrisk*_*i*_, to approximate the number of censored individuals on each time-interval *i*. We cannot identify the exact censoring pattern within each interval, and so we are forced to make an assumption. We have assumed that censoring occurs at a constant rate within each of the time intervals, which seems reasonable if the censoring pattern is non-informative (each subject has a censoring time that is statistically independent of their failure time).

**Figure 3:**
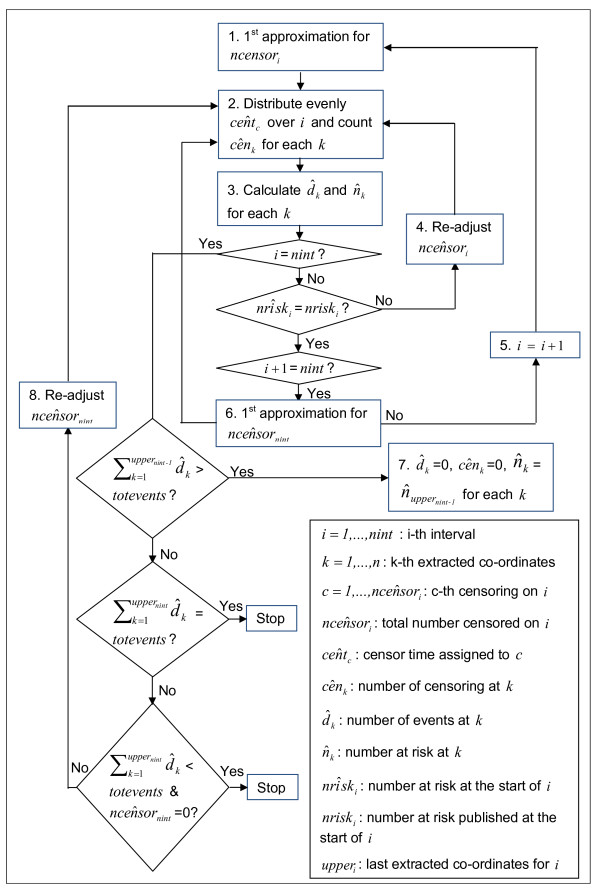
**Flowchart of the algorithm ('all information' case)**.

The algorithm is made up of the following steps (also illustrated in Figure 3).

STEP 1. We first form an initial guess for the number censored on interval *i*. If there were no individuals censored on interval *i *then the number at risk at the beginning of the following interval, $nrisk_{i+1}^{nocensor}$, would be the number at risk at the beginning of interval *i*, multiplied by the probability of experiencing the event at interval *i *conditional on being alive at the beginning of interval *i:*

$$nrisk_{i+1}^{nocensor}=nrisk_{i}*S_{lower_{i+1}}/S_{lower_{i}}$$

rounded to the nearest integer.

Our initial guess for the number censored on interval *i *is the difference between the reported number at risk at the beginning of interval *i *+ *1, nrisk*_*i*+1_, and the number at risk under no censoring:

(3) $$\begin{array}{c} nce\hat{n}sor_{i}=nrisk_{i+1}^{nocensor}-nrisk_{i+1}\\ nce\hat{n}sor_{i}=S_{lower_{i+1}}/S_{lower_{i}}*nrisk_{i}-nrisk_{i+1} \end{array}$$

STEP 2. We distribute the $c=1,\ldots ,nce\hat{n}sor_{i}$ censor times, $ce\hat{n}t_{c}$, evenly over interval *i*:

(4) $$\begin{array}{c} ce\hat{n}t_{c}=T_{lower_{i}}+c*\left(T_{lower_{i+1}}-T_{lower_{i}}\right)/\left(nce\hat{n}sor_{i}+1\right)\\ c=1,\ldots ,nce\hat{n}sor_{i} \end{array}$$

The number of censored observations between extracted KM co-ordinates *k *and *k *+ 1 is found by counting the number of estimated censor times, $ce\hat{n}t_{c}$, that lie between time *T*_*k *_and *T*_*k*+1_:

(5) $$cên_{k}=\overset{nce\hat{n}sor_{i}}{\underset{c=1}{\sum }}\left(ce\hat{n}t_{c}*I_{\left\{ce\hat{n}t_{c}\in \left[T_{k},T_{k+1}\right]\right\}}\right)$$

where $I_{\left\{ce\hat{n}t_{c}\in \left[T_{k},T_{k+1}\right]\right\}}$ is an indicator returning 1 if $ce\hat{n}t_{c}$ lies on the interval [*T*_*k*_, *T*_*k*+1_] and 0 otherwise.

STEP 3. The number of events, ${\hat{d}}_{k}$, at each extracted KM co-ordinate, *k*, and hence number of patients at risk at the next co-ordinate, ${\hat{n}}_{k+1}$, can then be calculated. Re-arranging Eq. 2, we obtain that ${\hat{d}}_{k}$ is equal to the number of patients at risk at the extracted KM co-ordinate, *k*, multiplied by one minus the probability of experiencing the event at the extracted KM co-ordinate, *k*, divided by ${Ŝ}_{last\left(k\right)}^{KM}$ the estimated KM survival probability at the previous co-ordinate where we estimate that an event occurred, *last*(*k*). The intervals of KM estimates are designed to be such that at least one event occurs at the start of each interval, but this is not necessarily the case for our extracted co-ordinates, and so we need to track the time of the last event:

$$last\left(k\right)=\left\{\begin{array}{cc} 1 & if\;k=1\\ k^{\prime } & otherwise \end{array}\right)$$

*where k' is such that *${\hat{d}}_{k^{\prime }}>0$

*but *${\hat{d}}_{j}=0$*for j *= *k' *+ 1,..., *k *- 1

Using eq.2, we have:

$${Ŝ}_{k}^{KM}=\left\{\begin{array}{cc} 1 & if\;k=1\\ {Ŝ}_{last\left(k\right)}^{KM}*\left(1-\frac{{\hat{d}}_{k}}{{\hat{n}}_{k}}\right) & otherwise \end{array}\right)$$

Therefore:

(6) $$\begin{array}{c} {\hat{d}}_{k}={\hat{n}}_{k}*\left(1-\frac{S_{k}}{{Ŝ}_{last\left(k\right)}^{KM}}\right)\\ k=lower_{i},\ldots ,upper_{i} \end{array}$$

rounded to the nearest integer.

The number of patients at risk at each extracted co-ordinate, *k*, is then obtained by using Eq.1:

(7) $$\begin{array}{c} {\hat{n}}_{k+1}={\hat{n}}_{k}-{\hat{d}}_{k}-cên_{k}\\ k=lower_{i},\ldots ,upper_{i} \end{array}$$

where at the start of the interval we set ${\hat{n}}_{lower_{i}}=nrisk_{i}$. This produces an estimated number at risk at the start of the following interval $nrîsk_{i+1}={\hat{n}}_{upper_{i}+1}$.

STEP 4. If $nrîsk_{i+1}\neq nrisk_{i+1}$ then we re-adjust the estimated number of censored observations in interval *i*, ncenŝor, by:

(8) $$ncenŝor_{i}=nce\hat{n}sor_{i}+\left({\hat{n}}_{upper_{i}+1}-nrisk_{i+1}\right)$$

We repeat steps 2-3 iteratively until estimated and published number at risk match (i.e. $nrîsk_{i+1}=nrisk_{i+1}$).

STEP 5. If *i *+ 1 is not the last interval, we repeat steps 1-4 for the following interval.

STEP 6. In published RCTs, there is generally no number at risk published at the end of the last interval, *nint*. We first assume that the number censored on the last interval is equal to the total number censored estimated prior to the last interval, $\overset{nint-1}{\underset{i=1}{\sum }}ncenŝor_{i}$, weighted by the remaining time relative to the time already elapsed, rounded to the nearest integer. But if this number was seen to be greater than the number of patients still at risk at the beginning of the last interval, this number at risk was chosen instead. This assumption is formally written in the equation below:

$$\begin{array}{c} nce\hat{n}sor_{nint}\\ =\text{min}\left(\frac{T_{upper_{nint}}-T_{lower_{nint}}}{T_{upper_{nint-1}}-T_{lower_{1}}}*\overset{nint-1}{\underset{i=1}{\sum }}nce\hat{n}sor_{i};nrisk_{nint}\right) \end{array}$$

And we run step 2-3.

STEP 7. We then use the reported total number of events, *totevents*. We calculate the estimated total number of events obtained by the beginning of the last interval, $\overset{upper_{nint-1}}{\underset{k=1}{\sum }}{\hat{d}}_{k}$. If this is greater or equal to *totevents *we assume that no more events or censoring occurs:

$$\begin{array}{c} {\hat{d}}_{k}=0,cên_{k}=0,\;{\hat{n}}_{k}=n_{upper_{nint-1}}\\ k=lower_{nint},\ldots ,upper_{nint} \end{array}$$

STEP 8. If $\overset{upper_{nint-1}}{\underset{k=1}{\sum }}{\hat{d}}_{k}$ is less than *totevents *we re-adjust the estimated number of censored observations in interval *nint*, $nce\hat{n}sor_{nint}$, by the difference in total number of events:

(9) $$nce\hat{n}sor_{nint}=nce\hat{n}sor_{nint}+\left(\overset{upper_{nint}}{\underset{k=1}{\sum }}{\hat{d}}_{k}-totevents\right)$$

We then re-run steps 2-3,8 for the last interval, *nint*, until the estimated total number of events, $\overset{upper_{nint-1}}{\underset{k=1}{\sum }}{\hat{d}}_{k}$, is equal to the reported total number of events, *totevents *or until the estimated total number of events is less than the reported total number of events but the total number of censoring in the last interval, $nce\hat{n}sor_{nint}$, becomes equal to zero.

#### Adjustments to the algorithm for the 'no numbers at risk' case

In this case there is only one interval *nint = 1*. We first assume that the total number censored is equal to zero and then we proceed as in step 8.

#### Adjustments to the algorithm for the 'no total events' case

In this case, we proceed as for the 'all information' case except that no re-adjustment using the total number of events can be done and we therefore stop at step 6.

#### Adjustment to the algorithm for the 'neither' case

When neither total number of events nor numbers at risk beyond the start of the study are reported, we assumed that there were no censored observations. This is a strong assumption, but as strong as any other assumption that we could make about the censoring without further information. Due to the lack of information, a lower quality of results is expected.

#### Obtaining the individual patient data (IPD) from the reconstructed Kaplan-Meier data

From our reconstructed Kaplan-Meier parameters ${\hat{d}}_{k},cên_{k},{\hat{n}}_{k}$ for each extracted KM co-ordinate *k *= 1,..., *N*, we can derive the IPD that would generate that data. This last piece of coding is in fact quite straightforward. Each time, that an event or a censoring is estimated, the corresponding time is recorded as well as an event indicator (one for event and zero for censoring).

### Evaluation of reproducibility and accuracy

Six pairs of Kaplan-Meier curves were used in the validation exercise. These were drawn from a subset of publications [12,27-29] that formed part of a look-back review of survival time analysis methods used in economic evaluations [13]. We carried out a reconstruction of twenty-two survival probabilities, seven median survival times, six hazard ratios and four standard errors of the log hazard ratios that were reported in these four publications. Each was reconstructed on two occasions by the same three observers. Two of the three observers were not involved in the development of the algorithm.

Reproducibility and accuracy of the method was evaluated for each of the 4 different levels of information ('all information', 'no numbers at risk', 'no total events' and 'neither'). To assess the differences between the reconstructed statistics and the original ones, the natural scale was used for the survival probabilities, while the log scale was used for medians, HRs and their uncertainties. Kaplan Meier curves and Cox HRs based on reconstructed data were estimated using the R routines survfit and coxph.

We fitted a standard two-way ANOVA with repeated measures to the differences between the reconstructed outcomes and the original outcomes, either on the natural or the log scale depending on the statistic considered. The components of variance were exemplar, observer, exemplar × observer interaction, and within-cell error. Because the p-value from the F-ratio test for the interaction was in all cases above 10%, we pooled the interaction term with the within-cell error term. The approach chosen is similar to what is referred to in engineering applications as 'gauge repeatability and reproducibility' [30,31].

The *reproducibility *represents the error if a single observer does a single reconstruction for a specified statistic. This was estimated as the sum of the within-observer and between-observer error. Monte Carlo simulation from the fitted ANOVA model was used to obtain the 95% confidence intervals around the standard deviations. The degrees of freedom for the within, the between and the outcome variations were assumed to follow chi-square distributions. To ensure robust inference, 150 000 samples of degrees of freedom were drawn from each of these distributions, i.e. for each source of variation. Then, the mean squares estimates were calculated, using the sum of squares obtained by the ANOVA and the sample obtained by the simulation, for each of the 150 000 samples and for each of the sources of variation. The corresponding 150 000 within, between and outcome standard deviations were subsequently estimated and we finally extracted the 2.5 and 97.5 percentiles to obtain the confidence intervals estimates.

To assess *accuracy *we examined the mean difference between the reconstructed statistics and the original ones. The resulting mean bias, or mean error (ME) reflects systematic over- or underestimation. The 95% confidence intervals are obtained directly from the estimation of the standard deviations given by the ANOVA. We also recorded absolute bias or mean absolute error (MAE). This ignores the direction of the errors and measures their magnitude, giving a measure of the absolute accuracy of the reconstructed outcomes. A simulation method was again used to obtain the 95% confidence intervals, which assumed that MEs were normally distributed. For each statistic, to ensure robust inference, 150 000 samples were drawn from the normal distribution with the observed mean and variance, as given by the ANOVA. We then calculated the corresponding 150 000 absolute values of these numbers and we finally extracted the 2.5 and 97.5 percentiles to obtain the confidence intervals estimates.

Finally we recorded the variation in the difference between reconstructed and original statistics that was due to the choice of exemplars, i.e. to the 22 survival probabilities, 7 medians, 6 HRs and 4 standard errors of the log HRs. This gives a further indication of the accuracy of the method.

## Competing interests

The authors declare that they have no competing interests.

## Authors' contributions

PG and NJW conceived the algorithm, with some substantial contribution from AEA and MJNMO. PG and AEA carried out the evaluation of accuracy and reproducibility of the algorithm, with some substantial contribution from NJM and MJNMO. All authors have been involved in drafting the manuscript. All authors have given final approval of the version to be published.

## Pre-publication history

The pre-publication history for this paper can be accessed here:

http://www.biomedcentral.com/1471-2288/12/9/prepub

## Supplementary Material

Additional file 1 **The algorithm (R coding).pdf**. Click here for file
