# Supplementary material for: Enhanced secondary analysis of survival data: reconstructing the data from published Kaplan-Meier survival curves
Source: BMC Med Res Methodol. 2012 Feb 1;12:9. doi: 10.1186/1471-2288-12-9 (PMC3313891; doi:10.1186/1471-2288-12-9)
Supplement: Additional file 1 — The algorithm (R coding).pdf. [file 1471-2288-12-9-S1.PDF]

```
#Algorithm to create a raw dataset from DigizeIt readings from a Kaplan-Meier curve

library("MASS")
library("splines")
library("survival")


###FUNCTION INPUTS
path<-"C:\\PHD\\algorithm\\reliability exercice\\"
digisurvfile<-"data initials study2 figA arm1 time1.txt"          #Input survival times from graph reading
nriskfile<-"nrisk study2 figA arm1 time1.txt"                    #Input reported number at risk
KMdatafile<-"KMdata study2 figA arm1 time1 ne.txt"               #Output file events and cens
KMdataIPDfile<-"KMdataIPD study2 figA arm1 time1 ne.txt"         #Output file for IPD
tot.events<-"NA"                    #tot.events = total no. of events reported. If not reported, then tot.events="NA"
arm.id<-1 #arm indicator
###END FUNCTION INPUTS

#Read in survival times read by digizeit
digizeit<- read.table(paste(path,digisurvfile,sep=""),header=TRUE)
t.S<-digizeit[,2]
S<-digizeit[,3]

#Read in published numbers at risk, n.risk, at time, t.risk, lower and upper
# indexes for time interval
pub.risk<-read.table(paste(path,nriskfile,sep=""),header=TRUE)
t.risk<-pub.risk[,2]
lower<-pub.risk[,3]
upper<-pub.risk[,4]
n.risk<-pub.risk[,5]
n.int<-length(n.risk)
n.t<- upper[n.int]

#Initialise vectors
arm<-rep(arm.id,n.risk[1])
n.censor<- rep(0,(n.int-1))
n.hat<-rep(n.risk[1]+1,n.t)
cen<-rep(0,n.t)
d<-rep(0,n.t)
KM.hat<-rep(1,n.t)
last.i<-rep(1,n.int)
sumdL<-0

if (n.int > 1){
#Time intervals 1,...,(n.int-1)
  for (i in 1:(n.int-1)){
        #First approximation of no. censored on interval i
        n.censor[i]<- round(n.risk[i]*S[lower[i+1]]/S[lower[i]]- n.risk[i+1])

        #Adjust tot. no. censored until n.hat = n.risk at start of interval (i+1)
        while((n.hat[lower[i+1]]>n.risk[i+1])||((n.hat[lower[i+1]]<n.risk[i+1])&&(n.censor[i]>0))){
                if (n.censor[i]<=0){
                        cen[lower[i]:upper[i]]<-0
                        n.censor[i]<-0
                }
                if (n.censor[i]>0){
                        cen.t<-rep(0,n.censor[i])
                        for (j in 1:n.censor[i]){
                                cen.t[j]<- t.S[lower[i]] +
                j*(t.S[lower[(i+1)]]-t.S[lower[i]])/(n.censor[i]+1)
                        }
                        #Distribute censored observations evenly over time. Find no. censored on each time interval.
                        cen[lower[i]:upper[i]]<-hist(cen.t,breaks=t.S[lower[i]:lower[(i+1)]],
                        plot=F)$counts
                }

                #Find no. events and no. at risk on each interval to agree with K-M estimates read from curves
                n.hat[lower[i]]<-n.risk[i]
        last<-last.i[i]
                for (k in lower[i]:upper[i]){
                        if (i==1 & k==lower[i]){
                                d[k]<-0
                                KM.hat[k]<-1
                        }
                        else {
                d[k]<-round(n.hat[k]*(1-(S[k]/KM.hat[last])))
```

```
                                                KM.hat[k]<-KM.hat[last]*(1-(d[k]/n.hat[k]))
                                        }
                                        n.hat[k+1]<-n.hat[k]-d[k]-cen[k]
        if (d[k] != 0) last<-k
                        }
                        n.censor[i]<- n.censor[i]+(n.hat[lower[i+1]]-n.risk[i+1])
        }
        if (n.hat[lower[i+1]]<n.risk[i+1]) n.risk[i+1]<-n.hat[lower[i+1]]
        last.i[(i+1)]<-last
  }
}

#Time interval n.int.
if (n.int>1){
#Assume same censor rate as average over previous time intervals.
n.censor[n.int]<- min(round(sum(n.censor[1:(n.int-1)])*(t.S[upper[n.int]]-
            t.S[lower[n.int]])/(t.S[upper[(n.int-1)]]-t.S[lower[1]])), n.risk[n.int])
}
if (n.int==1){n.censor[n.int]<-0}
if (n.censor[n.int] <= 0){
        cen[lower[n.int]:(upper[n.int]-1)]<-0
   n.censor[n.int]<-0
        }
if (n.censor[n.int]>0){
        cen.t<-rep(0,n.censor[n.int])
        for (j in 1:n.censor[n.int]){
                cen.t[j]<- t.S[lower[n.int]] +
                j*(t.S[upper[n.int]]-t.S[lower[n.int]])/(n.censor[n.int]+1)
        }
        cen[lower[n.int]:(upper[n.int]-1)]<-hist(cen.t,breaks=t.S[lower[n.int]:upper[n.int]],
                              plot=F)$counts
}

#Find no. events and no. at risk on each interval to agree with K-M estimates read from curves
n.hat[lower[n.int]]<-n.risk[n.int]
last<-last.i[n.int]
for (k in lower[n.int]:upper[n.int]){
                if(KM.hat[last] !=0){
           d[k]<-round(n.hat[k]*(1-(S[k]/KM.hat[last])))} else {d[k]<-0}
        KM.hat[k]<-KM.hat[last]*(1-(d[k]/n.hat[k]))
        n.hat[k+1]<-n.hat[k]-d[k]-cen[k]
        #No. at risk cannot be negative
        if (n.hat[k+1] < 0) {
                n.hat[k+1]<-0
                cen[k]<-n.hat[k] - d[k]
        }
        if (d[k] != 0) last<-k
}

#If total no. of events reported, adjust no. censored so that total no. of events agrees.
if (tot.events != "NA"){
        if (n.int>1){
        sumdL<-sum(d[1:upper[(n.int-1)]])
        #If total no. events already too big, then set events and censoring = 0 on all further time intervals
        if (sumdL >= tot.events){
                d[lower[n.int]:upper[n.int]]<- rep(0,(upper[n.int]-lower[n.int]+1))
                cen[lower[n.int]:(upper[n.int]-1)]<- rep(0,(upper[n.int]-lower[n.int]))
                n.hat[(lower[n.int]+1):(upper[n.int]+1)]<- rep(n.risk[n.int],(upper[n.int]+1-lower[n.int]))
        }
                }
        #Otherwise adjust no. censored to give correct total no. events
        if ((sumdL < tot.events)|| (n.int==1)){
                sumd<-sum(d[1:upper[n.int]])
                while ((sumd > tot.events)||((sumd< tot.events)&&(n.censor[n.int]>0))){
                        n.censor[n.int]<- n.censor[n.int] + (sumd - tot.events)
                        if (n.censor[n.int]<=0){
                                cen[lower[n.int]:(upper[n.int]-1)]<-0
                                n.censor[n.int]<-0
                        }
                        if (n.censor[n.int]>0){
                                cen.t<-rep(0,n.censor[n.int])
                                for (j in 1:n.censor[n.int]){
                                        cen.t[j]<- t.S[lower[n.int]] +
                            j*(t.S[upper[n.int]]-t.S[lower[n.int]])/(n.censor[n.int]+1)
                                }
```

```
                                        cen[lower[n.int]:(upper[n.int]-1)]<-hist(cen.t,breaks=t.S[lower[n.int]:upper[n.int]],
                                      plot=F)$counts
                                        }
                                        n.hat[lower[n.int]]<-n.risk[n.int]
                                        last<-last.i[n.int]
                                        for (k in lower[n.int]:upper[n.int]){
                                                d[k]<-round(n.hat[k]*(1-(S[k]/KM.hat[last])))
                                                KM.hat[k]<-KM.hat[last]*(1-(d[k]/n.hat[k]))
                                                if (k != upper[n.int]){
                                                        n.hat[k+1]<-n.hat[k]-d[k]-cen[k]
                                                        #No. at risk cannot be negative
                                                        if (n.hat[k+1] < 0) {
                                                                n.hat[k+1]<-0
                                                                cen[k]<-n.hat[k] - d[k]
                                                        }
                                                }
                                                if (d[k] != 0) last<-k
                                        }
                        sumd<- sum(d[1:upper[n.int]])
                        }
        }
}


write.table(matrix(c(t.S,n.hat[1:n.t],d,cen),ncol=4,byrow=F),paste(path,KMdatafile,sep=""),sep="\t")

### Now form IPD ###
#Initialise vectors
t.IPD<-rep(t.S[n.t],n.risk[1])
event.IPD<-rep(0,n.risk[1])
#Write event time and event indicator (=1) for each event, as separate row in t.IPD and event.IPD
k=1
for (j in 1:n.t){
        if(d[j]!=0){
                t.IPD[k:(k+d[j]-1)]<- rep(t.S[j],d[j])
                event.IPD[k:(k+d[j]-1)]<- rep(1,d[j])
                k<-k+d[j]
        }
}
#Write censor time and event indicator (=0) for each censor, as separate row in t.IPD and event.IPD
for (j in 1:(n.t-1)){
        if(cen[j]!=0){
                t.IPD[k:(k+cen[j]-1)]<- rep(((t.S[j]+t.S[j+1])/2),cen[j])
                event.IPD[k:(k+cen[j]-1)]<- rep(0,cen[j])
                k<-k+cen[j]
        }
}

#Output IPD
IPD<-matrix(c(t.IPD,event.IPD,arm),ncol=3,byrow=F)
write.table(IPD,paste(path,KMdataIPDfile,sep=""),sep="\t")

#Find Kaplan-Meier estimates
IPD<-as.data.frame(IPD)
KM.est<-survfit(Surv(IPD[,1],IPD[,2])~1,data=IPD,type="kaplan-meier",)
KM.est
summary(KM.est)


#Find Cox hazard ratio
data0<- read.table("C:\\PHD\\algorithm\\reliability exercice\\KMdataIPD study2 figA arm0 time2 no.txt",header=TRUE)
data1<- read.table("C:\\PHD\\algorithm\\reliability exercice\\KMdataIPD study2 figA arm1 time2 no.txt",header=TRUE)
data<-merge(data0,data1,all=T)
cox<-coxph(formula = Surv(data[,1], data[,2]) ~ data[,3], data=data)
summary(cox)
```
